# Enhanced Aquathermolysis of Water–Heavy Oil–Ethanol Catalyzed by B@Zn(II)L at Low Temperature

**DOI:** 10.3390/molecules29092057

**Published:** 2024-04-29

**Authors:** Zhe Shen, Xiangqing Fang, Wenbo He, Le Zhang, Yongfei Li, Guobin Qi, Xin Xin, Bin Zhao, Gang Chen

**Affiliations:** 1 The Institute of Energy and Architecture, Xi’an Aeronautical Institute, Xi’an 710077, China; 2 Shaanxi Province Key Laboratory of Environmental Pollution Control and Reservoir Protection Technology of Oilfields, Xi’an Shiyou University, Xi’an 710065, China; 3 Engineering Research Center of Oil and Gas Field Chemistry, Universities of Shaanxi Provence, Xi’an Shiyou University, Xi’an 710065, China; 4 CCDC Changqing Downhole Technology Company, Xi’an 710060, China; 5 Department of Crop Soil Sciences, Washington State University, Pullman, WA 99163, USA; 6 Department of Statistics, North Dakota State University, Fargo, ND 58102, USA

**Keywords:** heavy oil, catalytic aquathermolysis, viscosity reduction, clay, bentonite complex catalyst, ethanol

## Abstract

In order to study the synergistic effects of exogenous catalysts and in situ minerals in the reservoir during heavy oil aquathermolysis, in this paper, a series of simple supported transition metal complexes were prepared using sodium citrate, chloride salts and bentonite, and their catalytic viscosity reduction performances for heavy oil were investigated. Bentonite complex catalyst marked as B@Zn(II)L appears to be the most effective complex. B@Zn(II)L was characterized by scanning electron microscopy (SEM), Fourier-Transform Infrared (FTIR) spectroscopy, thermo-gravimetric analysis (TGA) and N2 adsorption–desorption isotherms. Under optimized conditions, the viscosity of the heavy oil was decreased by 88.3%. The reaction temperature was reduced by about 70 °C compared with the traditional reaction. The results of the group composition analysis and the elemental content of the heavy oil indicate that the resin and asphaltene content decreases, and the saturated and aromatic HC content increases. The results of TGA and DSC of the heavy oil show that the macromolecular substances in the heavy oil were cracked into small molecules with low boiling points by the reaction. GC-MS examination of water-soluble polar compounds post-reaction indicates that B@Zn(II)L can diminish the quantity of polar substances in heavy oil and lower the aromatic nature of these compounds. Thiophene and quinoline were utilized as model compounds to investigate the reaction mechanism. GC-MS analysis revealed that C-C, C-N and C-S bonds were cleaved during the reaction, leading to a decrease in the viscosity of heavy oil.

## 1. Introduction

Heavy oil contains long-chain cyclic fused ring compounds such as resins and asphaltenes, which contribute to its high viscosity and low fluidity [1]. Functional groups like hydroxyl, amino, and carboxyl groups can lead to the creation of hydrogen bonds in resin and asphaltenes. The high melting and boiling temperatures, as well as the high viscosity of crude oil, are attributed to the molecular hydrogen bonds formed between resin and asphaltene molecules [2,3,4,5]. In addition, due to the complex environment of heavy oil reservoirs, great difficulties are encountered in the process of oil exploration and transportation [6]. Using viscosity reduction technology, heavy oil can be converted into thin oil, which improves the utilization rate of heavy oil and reduces oil consumption. As the most promising technology for heavy oil extraction, catalytic hydrothermal cracking technology is currently being developed [7]. According to Hyne in 1982, metals can speed up hydrothermal decomposition. This reaction has subsequently been studied in the context of several catalysts [8,9,10]. Hyne et al. also used thiophene and tetrahydrothiophene as models to study the mechanism of hydrothermal catalytic cracking reaction. The breakage of C-S bonds is primarily responsible for the heavy oil’s decreased viscosity, and transition metal compounds can clearly act as catalysts in this process. The complexation of the metal atoms with organic sulfur can be better influenced by the ligands, which can result in the breaking of the C-S bond and the beginning of the acid polymerization and water–gas shift processes. In this approach, the heavy oil’s viscosity is decreased, enhancing its quality [11,12,13]. Steam’s energy causes certain large molecules to be pyrolyzed into smaller molecules throughout the reaction process, and catalysis may remove some heteroatoms [14,15]. Therefore, the ligand’s presence may increase the catalyst’s catalytic action. Furthermore, the clay’s surface is abundant in hydroxyl groups (-OH). When the catalyst is added, the transition metal salt can be adsorbed on the clay surface via ion exchange, hydrogen bonding, and other supramolecular processes, generating a complex. The transition metal salt may also decompose under the clay’s catalytic effect to produce additional active components [16,17]. Furthermore, the presence of clay-loaded metal complexes can catalyze the production of hydrogen via alcohol reforming: taking methanol as an example, at a certain temperature and pressure, in the presence of water and a catalyst, cracking and carbon monoxide conversion reactions occur, generating H_2_ and CO_2_ [18,19]. Maintaining a heavy oil–water–alcohol hydrogen-rich reaction system will improve the viscosity reduction efficiency of heavy oil during hydrothermal cracking.

In this study, we used the reservoir material clay bentonite as a substrate to produce a variety of supported complex catalysts. The core metal ions were provided by seven transition metal chlorides, and sodium citrate functioned as a ligand and calcium bentonite as a carrier. The hydrogen-rich reaction employed the bentonite-supported metal complex catalysts to successfully lower the viscosity of the oil, water, and ethanol mixture. Thermogravimetric analysis (TGA), differential scanning calorimetry (DSC), and elemental analysis (EL) were used to examine the heavy oil both before and after the procedure. Gas chromatography (GC) was used to determine the concentration of saturated hydrocarbons (HC) both before and after the reaction. Additionally, the polar chemicals dissolved in the water following the heavy oil reaction were assessed using Gas Chromatography–Mass Spectrometry (GC-MS).

## 2. Results and Discussion

### 2.1. Aquathermolysis Reaction Condition Screening

**Figure 1:**
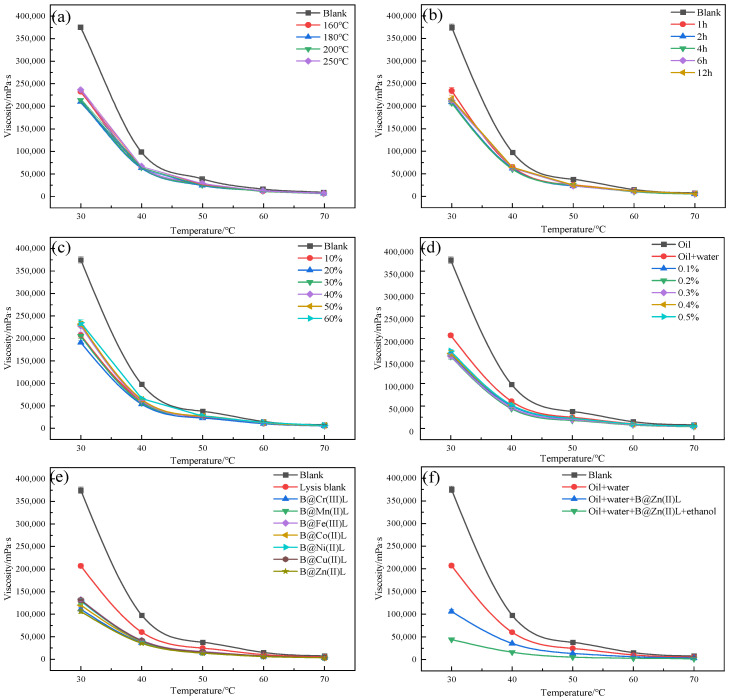
Effects of reaction temperature (**a**), reaction duration (**b**), mass ratio of water to oil (**c**), calcium soil addition (**d**), different catalysts (**e**), and addition of ethanol (**f**) on the temperature-dependent viscosity of heavy oil after thermolysis.

Reaction temperature is the most important factor in the process of aquathermolysis of heavy oil, so this was investigated first, with a reaction duration of 2 h. The results are shown in Figure 1a, and the blank is the oil sample without any treatment. From Figure 1a, it is clear that the viscosity drops significantly for all the temperatures tested (ranging from 160 to 250 °C), with the greatest decrease at 180–200 °C. The decrease is less at 160 and 250 °C—the former is due to the reaction proceeding to a lesser extent at lower temperatures, while the latter is attributed to polymerization reactions occurring to a greater extent at high temperatures, causing an increase in the viscosity of the heavy oil and formation of solid coke. Obviously, the reaction temperature of 180 °C is lower than that of conventional reactions [20]. After this, the influence of the reaction time on the hydrothermal cracking reaction of the oil sample was investigated at a reaction temperature of 180 °C, and the results are shown in Figure 1b. It seems that the reaction time of 2 h is enough to reduce the viscosity of the heavy oil; the long reaction time does not result in significant changes in viscosity. However, in specifying optimal reaction conditions, we chose 4 h as the reaction duration to be on the safe side. In addition, in order to determine the effect on hydrothermal decomposition of the amount of water added, the mass ratio of water to oil was investigated at the optimal reaction temperature of 180 °C and optimal reaction time of 4 h, and the results are shown in Figure 1c. Figure 1c shows that the viscosity decreased most effectively with the addition of an amount of water equal to 20% of the mass of the heavy oil. Therefore, we chose 20% as the optimal amount of water to be added to the oil sample. Calcium-based bentonite was used as the carrier of the catalyst; the amount of catalyst to be added was determined by investigating the addition of variable amounts of calcium soil under the optimal reaction conditions already derived (180 °C, 4 h, 20% water). The viscosity after the hydrothermal cracking reaction is shown in Figure 1d. Figure 1d demonstrated that addition of calcium soil in an amount of 0.2% of the mass of the heavy oil led to the highest viscosity reduction for the oil sample. Therefore, the amount of catalyst to be added was set at 0.2%. With the reaction conditions thus specified, the catalytic aquathermolysis of the crude oil was investigated with the addition of the seven transition-metal-based catalysts (in 0.2% amounts), and the results are exhibited in Figure 1e.

All seven catalysts were found to be effective in the process, with B@Zn(II)L being the most effective, resulting in a viscosity drop from 375,000 to 148,000 mPa·s. This amounts to a viscosity reduction rate of 60.53%. Therefore, B@Zn(II)L was used in the rest of this study. Figure 1f shows that the viscosity reduction rate reaches 88.3% after the addition of ethanol (in an amount equal to 30% of the heavy oil mass), which suggests that ethanol, as a hydrogen source, is effective for reducing the viscosity of heavy oil.

**Figure 2:**
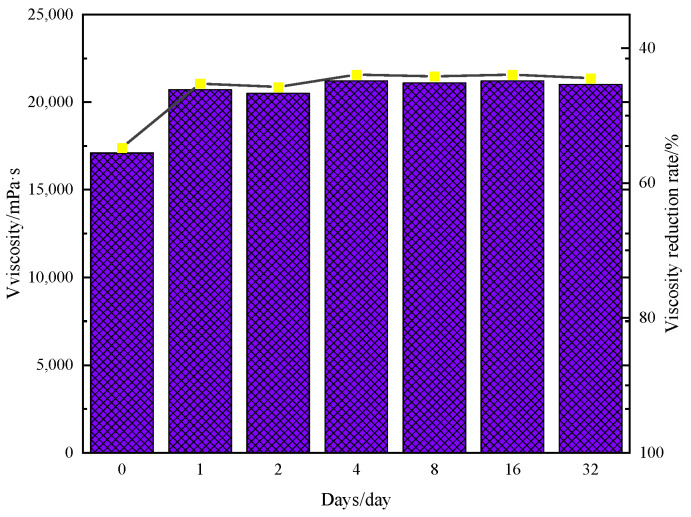
Dependence of heavy oil viscosity on time after reaction with B@Zn(II)L.

The viscosity of the crude oil was assessed following its reaction with water, B@Zn(II)L, and ethanol and subsequent storage at 50 °C for a period ranging from 0 to 32 days. The results are displayed in Figure 2. The oil’s viscosity marginally improves after 1 day and then remains quite stable. These data suggest that the alteration in viscosity is mostly caused by chemical reactions rather than a physical process. The chemical alterations and reaction mechanism will be examined and analyzed in the upcoming study.

### 2.2. Characterization of the Crude Oil

Table 1 shows the chemical makeup of the heavy oil before and after the process. It is evident that the quantity of resin and asphaltene dropped, while the quantity of saturated hydrocarbons and aromatic hydrocarbons increased following the reaction. The decrease in asphaltene means that this component was depolymerized to give other components [21,22,23].

Table 2 shows the elemental analysis findings of oil samples in different reaction conditions. The analysis shows decreases in the levels of nitrogen and sulfur in the oil samples after the reaction. During aquathermolysis, the cleavage of C-R (R = S, N, O) bonds generates numerous free radicals. Free radicals can react with water and/or ethanol molecules, resulting in a decrease in asphaltene content and improving the quality of heavy oil [24].

**Figure 3:**
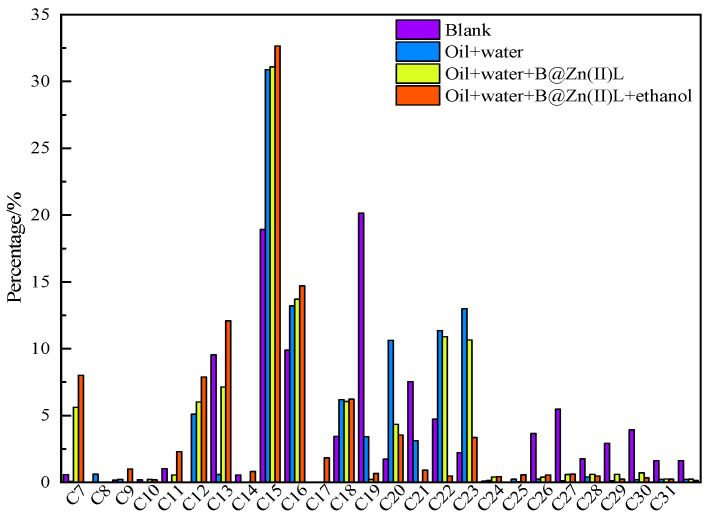
Gas chromatograph analysis of saturated hydrocarbons in the crude oil before and after the reaction.

Gas chromatography was employed to assess the carbon number distribution in saturated hydrocarbons, as shown in Figure 3. This can reveal changes in the carbon number distribution of saturated hydrocarbons in oil samples under different reaction conditions. Figure 3 demonstrates that the addition of a catalyst and the reaction with ethanol lead to a decrease in the quantity of high-carbon-number chains, while the carbon numbers C7–C16 increase, aligning with the findings of the group composition analysis.

**Figure 4:**
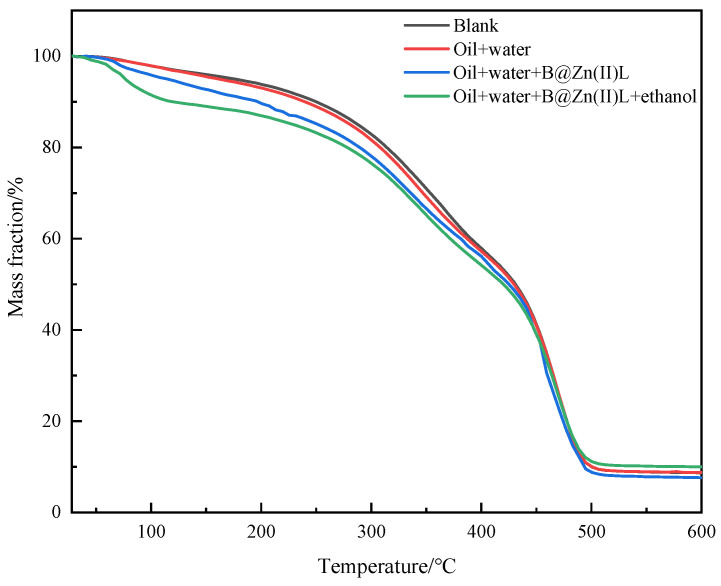
The TG curves of the crude oil before and after aquathermolysis.

Thermogravimetric analysis (TGA) indicates alterations in the volatility of the components due to the reaction, as illustrated in Figure 4. Similar patterns are shown by the curves of crude oil treated with different reaction settings. Introducing B@Zn(II)L and ethanol considerably shifts the weight loss curve to the left and increases the weight loss rate of the oil sample. The results show that the large molecules in the heavy oil were broken down into smaller molecules with a lower boiling point during the reaction.

**Figure 5:**
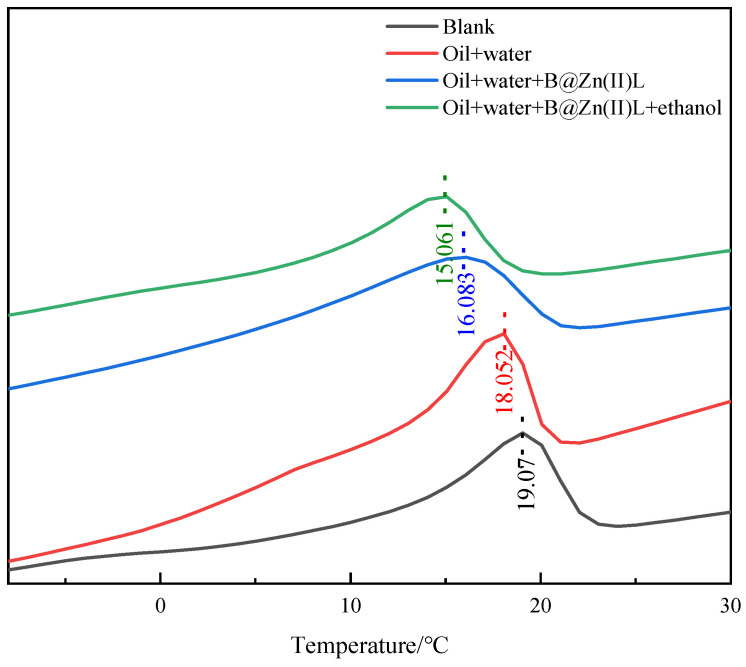
DSC of the crude oil before and after aquathermolysis.

Differential scanning calorimeter (DSC) analysis was used to investigate the wax production process in the crude oil under various reaction circumstances (Figure 5). The wax precipitation peak of the crude oil occurs at 19.07 °C. The utilization of B@Zn(II)L and ethanol in the reaction resulted in a decrease in the wax precipitation temperature from 19.07 °C to 15.06 °C, indicating the breakdown of heavier components in the oil sample into lighter components. Furthermore, certain dense components were dissolved in the less dense components. The alteration in the components’ balance decelerated the wax crystal precipitation and lowered the wax precipitation temperature of the oil sample.

### 2.3. Analysis of Polar Substances Dissolved in Water after Heavy Oil Reaction

**Figure 6:**
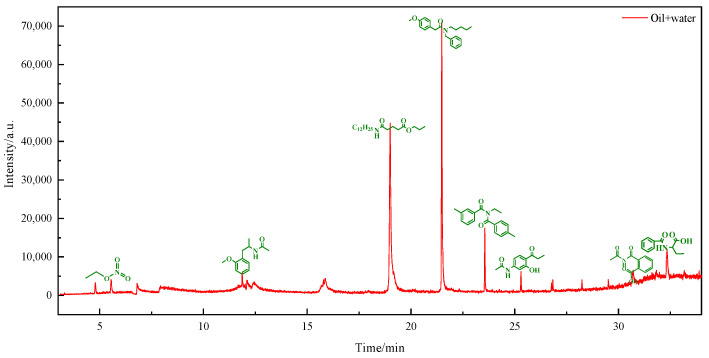
Analyses of polar substances dissolved in water following their reaction with water via GC/MS.

**Figure 7:**
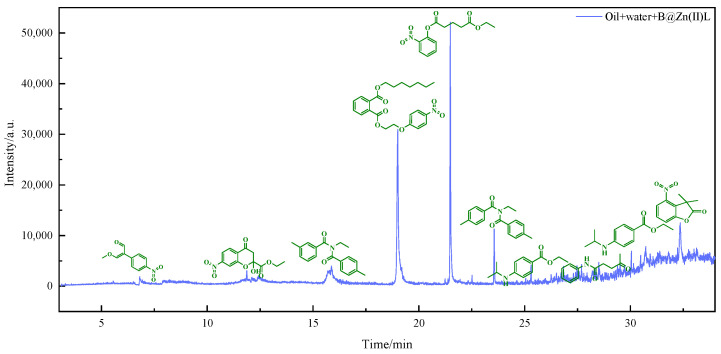
Water and B@Zn(II)L reactions with polar substances in water via GC/MS.

**Figure 8:**
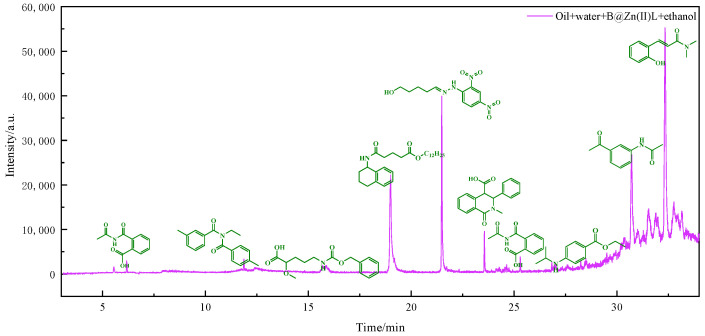
GC/MS analysis was conducted on polar compounds dissolved in water following their interaction with water, B@Zn(II)L, and ethanol.

The viscosity reduction mechanism of heavy oil was further elucidated by analyzing the polar components dissolved in water after reaction using GC-MS. Figure 6, Figure 7 and Figure 8 display the outcomes of the reaction using water, water and B@Zn(II)L, and water, B@Zn(II)L, and ethanol, respectively. Figure 6, Figure 7 and Figure 8 show that a significant quantity of polar organic matter is dissolved in the water after the reaction. Aromatic ring-containing compounds are removed from heavy oil, leading to a decrease in the heavy oil’s viscosity. Comparing the figures reveals that Figure 6 contains fewer polar compounds, whereas Figure 8 has the most polar chemicals. The presence of the catalyst and ethanol can decrease the quantity of polar compounds in heavy oil. In particular, this includes the removal of aromatic polar substances; as these make a significant contribution to the viscosity of the heavy oil, their removal also contributes to viscosity reduction.

### 2.4. Characterizations of Catalyst

**Figure 9:**
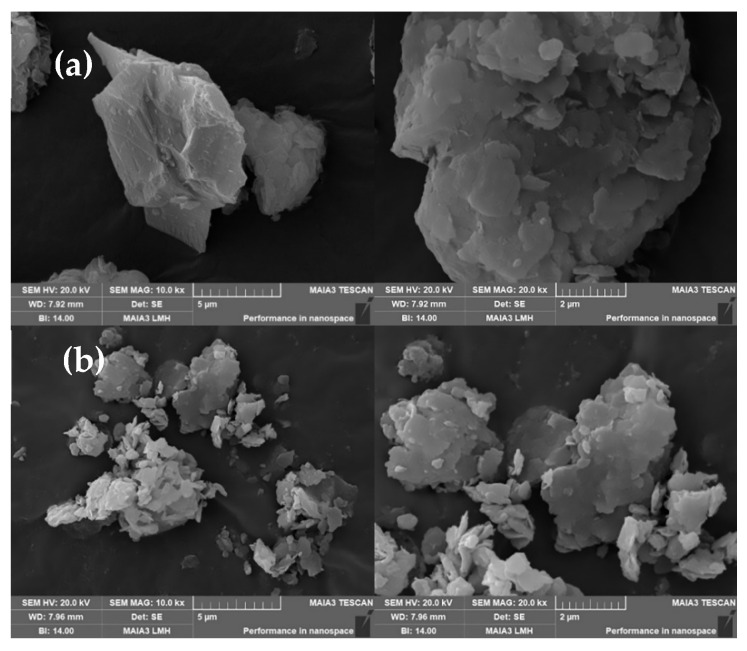
SEM of B (**a**) and SEM of B@Zn(II)L (**b**).

Scanning electron microscopy (SEM) was used to analyze the morphology of bentonite (B) and B@Zn(II)L. Figure 9a illustrates that bentonite has bigger particles, approximately 5 μm in size, and a compact interlayer distribution. Figure 9b displays the lamellar and petal-like morphology of B@Zn(II)L, with a particle size of around 600 nm. The size of the B@Zn(II)L particle has decreased, resulting in enhanced dispersibility and a larger surface area. This increases the contact area between the crude oil and the metal by firmly adsorbing it onto the surface and inner wall of the pore.

**Figure 10:**
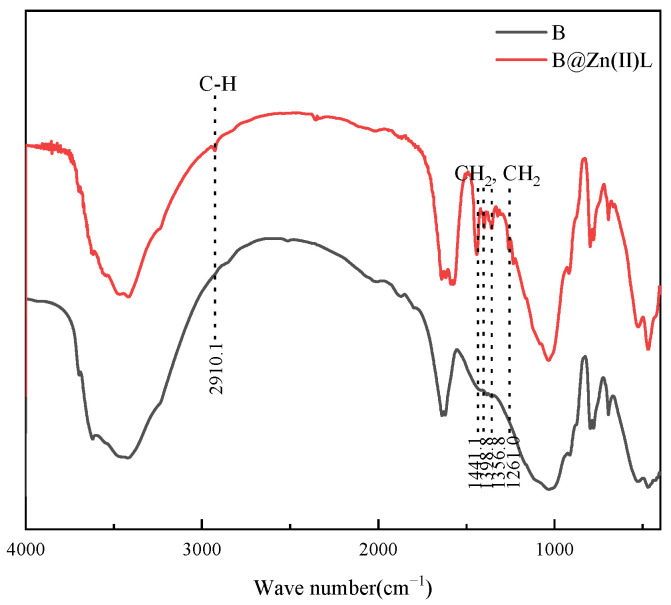
FT-IR spectra of B and B@Zn(II)L.

Furthermore, B and B@Zn(II)L were analyzed via infrared spectroscopy; the results are shown in Figure 10. The new band occurring at 2910.1 cm^−1^ is attributed to the stretching vibrations of the C-H groups. B@Zn(II)L also has bands at 1261.0, 1356.8, 1398.8, and 1441.1 cm^−1^, which are attributed to -CH_2_ deformation vibrations [25].

The presence of these hydrocarbon signals confirms the presence of the Zn(II)L complex in the bentonite.

**Figure 11:**
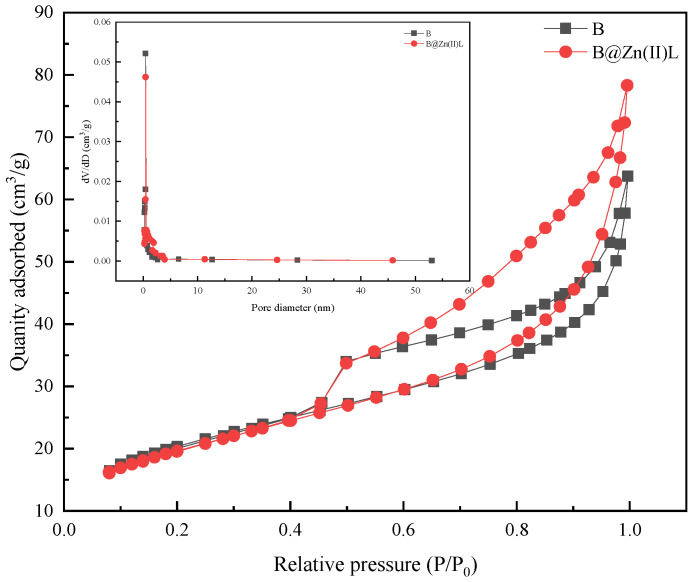
Nitrogen adsorption–desorption isotherms and pore size distribution of B and B@Zn(II)L.

N_2_ adsorption–desorption isotherms of B and B@Zn(II)L were analyzed using the Brunauer–Emmett–Teller (BET) method. The parameters are presented in Table 3, and the N_2_ adsorption–desorption isotherm of the supported catalyst exhibits a type IV isotherm, as shown in Figure 11. Table 3 shows that the pore volume and pore size of B@Zn(II)L increased compared to the original bentonite, resulting in the enhanced absorption capacity of the supported catalyst B@Zn(II)L [26].

**Figure 12:**
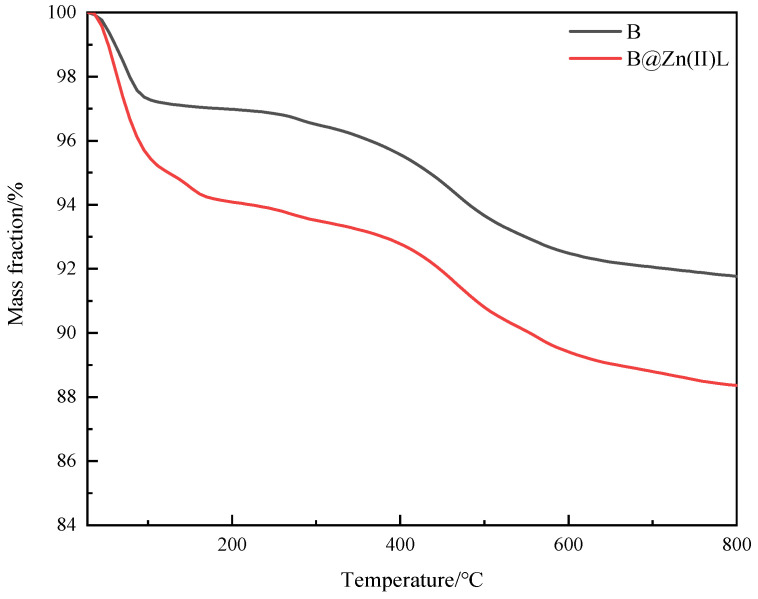
Thermogravimetric curves of B and B@Zn(II)L.

The catalyst’s thermal stability was examined using thermogravimetric measurement of B@Zn(II)L (Figure 12). Figure 12 displays a notable decrease in the mass of B@Zn(II)L from 30 °C to 100 °C as a result of water loss. The mass loss rate of B@Zn(II)L dropped at higher temperatures but remained higher than that of B, suggesting that the thermal stability of the supported catalyst B@Zn(II)L is lower than that of the unmodified clay.

### 2.5. Reaction Mechanism

**Figure 13:**
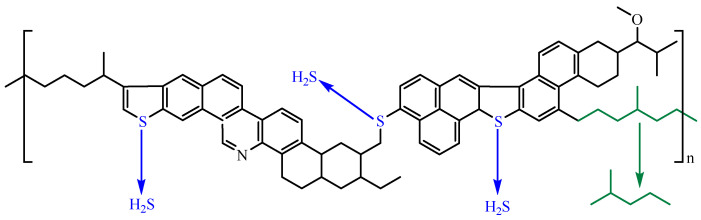
The structures of resin and asphaltene model compounds.

**Figure 14:**
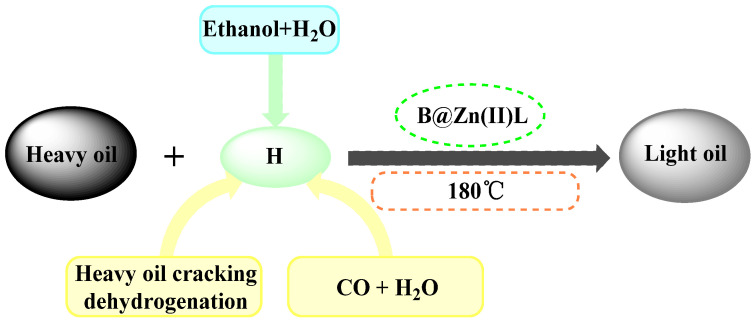
Reaction mechanism.

Resin and asphaltene have many functional groups that can create hydrogen bonds. Figure 13 illustrates the composition of resin and asphaltene model compounds. Hydrothermal cracking can induce hydrogenation, bond breakage, ring opening reactions, and other chemical transformations. Adding B@Zn(II)L and ethanol to the crude oil phase decreases the hydrogen bonds between the resin and the asphaltene [27]. In this investigation, we used a solution containing 0.2% B@Zn(II)L and 30% ethanol, heated at 180 °C for 4 h. Our hypothesis is that metal ions facilitate acid polymerization and water–gas shift reactions through interactions with sulfur atoms. These reactions are triggered by breaking the C-S bond, leading to the creation of free radicals. This inhibits the clumping together of resin and asphaltene molecules in the crude oil and the creation of interconnected structures, leading to a decrease in the viscosity of the crude oil; the viscosity reduction process is seen in Figure 14.

**Figure 15:**
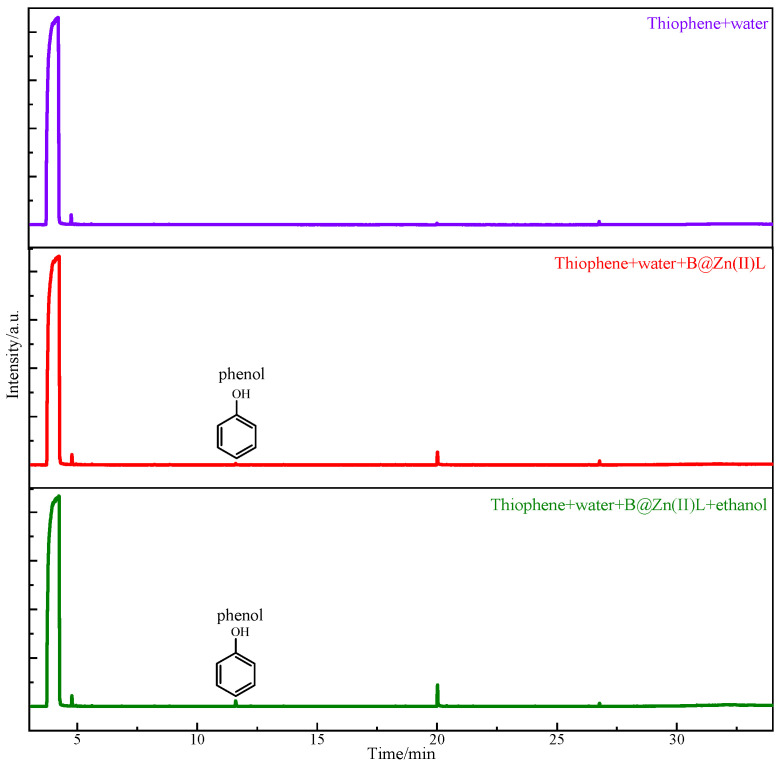
Chromatogram of thiophene after reaction under different conditions.

**Figure 16:**
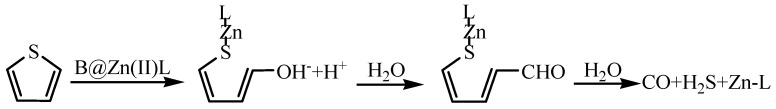
Reaction mechanism of thiophene.

**Figure 17:**
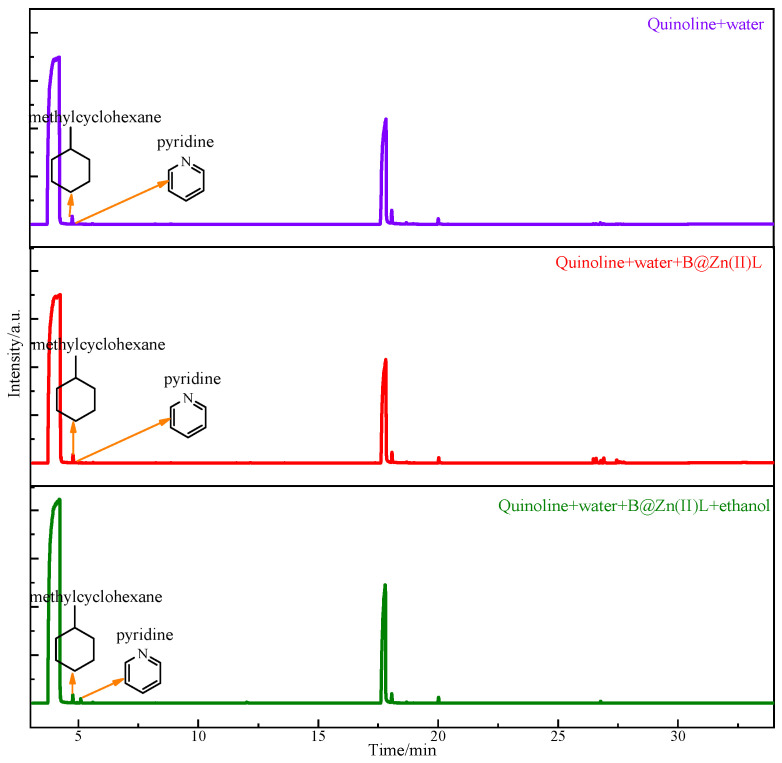
Chromatogram of quinoline after reaction under different conditions.

To investigate the catalytic viscosity reduction mechanism of B@Zn(II)L, two model compounds, thiophene and quinoline, were employed to mimic the reaction of common heavy oil compounds. The solutions post-reaction under various circumstances were examined using GC-MS. Figure 15 shows that phenol was detected in the products resulting from the reaction of thiophene with water, B@Zn(II)L, and ethanol [28,29]. The catalyst undergoes an oxidation–reduction reaction, resulting in the production of CO and H_2_S, as depicted in Figure 16. The reaction resulted in the cleavage of the C-S bond, leading to a decrease in the viscosity of the heavy oil [18,19]. Figure 17 displays the results of the reaction of quinoline with water, B@Zn(II)L, and ethanol, resulting in the presence of methylcyclohexane and pyridine.

**Figure 18:**
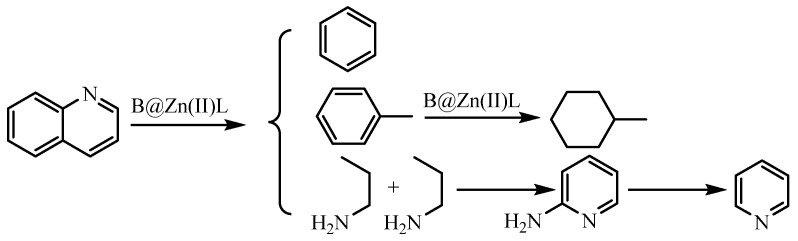
Reaction mechanism of quinoline.

Hydrogen breaking the C-C and C-N bonds in quinoline produces benzene, toluene, and propylamine. Methylcyclohexane is produced by hydrogenating toluene, while pyridine is made through the cyclization and deamination of two propylamine molecules, as shown in Figure 18. The reaction with B@Zn(II)L and ethanol cleaves the C-C, C-N, and C-S bonds, resulting in a significant reduction in the viscosity of the heavy oil.

## 3. Materials and Methods

### 3.1. Materials

Petroleum ether, toluene, n-heptane, and 100% ethanol were obtained from Xi’an Chemical Reagent Co., Ltd, Xi’an City, Shaanxi Province, China. at analytical reagent grade. All reagents in this study were purchased and used without further purification. The crude oil originated from Tanghe Oilfield in China. The oil sample’s principal elemental composition was determined using an elemental analyzer (Vario EL cube, Elementar Analysensyteme GmbH, Berlin, Germany). The calcium bentonite was obtained from Fengyun Chemical Co., Ltd. in Xi’an, China.

### 3.2. Synthesis and Characterization of the Catalyst

**Figure 19:**
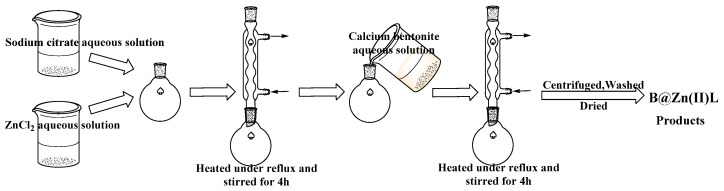
Preparation of B@Zn(II)L.

Zinc chloride (ZnCl_2_) and sodium citrate were dissolved in distilled water at a 1:1 molar ratio and heated under reflux for 4 h to generate Zn(II)L. Subsequently, 4 g of calcium bentonite was introduced into the mixture, followed by a 4-h reflux, centrifugation at 4000 revolutions per minute, and rinsing with distilled water. The goods were then dried for a long time at 70 °C. Figure 19 shows the schematic diagram of the B@Zn(II)L synthesis process. The B@Zn(II)L was examined via scanning electron microscopy (SEM, JSM-6390A, JEOL, Showima City, Tokyo, Japan), Fourier-Transform Infrared spectroscopy (FTIR, Nicolet 5700, Waltham, MA, USA), thermogravimetric analysis (TGA), and N_2_ adsorption–desorption isotherms. The manufacture of the additional catalysts (B@Cr(Ⅲ)L, B@Mn(Ⅱ)L, B@Fe(Ⅲ)L, B@Co(Ⅱ)L, B@Ni(Ⅱ)L, B@Cu(Ⅱ)L, B@Zn(Ⅱ)L) followed the same procedure, but with varying chloride salts.

### 3.3. Catalysis of Complex Used in Aquathermolysis Reaction of the Heavy Oil

The experiments were conducted by introducing 35 g of heavy oil sample, along with varying quantities of water, catalyst, and ethanol, into the high-temperature reactor to study the impact of reaction temperature, reaction time, catalyst dosage, and the amount of water and ethanol added. The liquid was then heated to start the aquathermolysis reaction. Upon the completion of the experiment, the heating process was halted, and the mixture was then cooled down to 40 °C for testing and analysis.

### 3.4. Evaluation and Analysis of the Heavy Oil

The viscosity of the heavy oil was recorded using a BROOKFIELD DV-II+ programmable viscometer at different temperatures. The viscosity reduction ratio Δη (in %) was calculated as follows [10]:

Δη = [(η_0_ − η_1_)/η_0_] × 100 (1)

The symbol Δη represents the viscosity reduction, η_0_ represents the initial viscosity of the oil (mPa·s), and η_1_ represents the viscosity of the oil after the reaction (mPa·s) [10,30].

Furthermore, the heavy oil underwent additional analysis to examine any structural modifications and group composition. Four categories of chemicals, saturated hydrocarbons, aromatic hydrocarbons, resins, and asphaltenes (SARA), were isolated using column chromatography following the China Petroleum industry standard SY/T 5119 [31]. We analyzed the heavy oil’s elemental composition before and after the reaction using an EL-2 elemental analyzer. GC analysis of the saturated hydrocarbon was performed before and after the reaction using an HP-GC6890 GC analyzer [32]. The oil sample was mixed with water, reservoir minerals, external catalysts, and a hydrogen donor in a reactor operating at high temperature and pressure. The reaction occurred at a temperature of 200 °C for a duration of 4 h.

### 3.5. Thermogravimetric Analysis (TGA)

In order to determine the temperature resistance of the synthesized catalyst, a thermogravimetric analysis (TGA) was conducted with a TGA/SDTA851 instrument from METTLERTOLEDO, covering a temperature range extending from 25 to 600 °C, with a heating rate of 10 °C/min. The tests were conducted in a nitrogen (N_2_) atmosphere with a flow rate of 20 mL/min, using silicon dioxide (SiO_2_) crucibles [24].

### 3.6. Differential Scanning Calorimetry (DSC) Analysis

The wax appearance temperature in the crude oil with each flow improver was determined using DSC analysis following standard SY/T 0545-2012. The DSC test was performed with a Mettler-Toledo DSC822e instrument from Switzerland under a N_2_ atmosphere with a flow rate of 70 mL/min. The test encompassed a temperature range spanning from −20 to 50 °C, with a heating speed of 10 °C/min [25].

### 3.7. GC-MS Analysis

After hydrothermally cracking the heavy oil under various conditions, the solid impurities in the water phase were isolated and moved to a round-bottomed flask. The polar compounds remaining after removing the water were dissolved in 100% ethanol to prepare them for GC-MS analysis. The solution was analyzed using a 7890A-5975C apparatus with hydrogen as the carrier gas flowing at a rate of 25 mL/min. The material was analyzed using the DRS chemical database. K. Chao provides a detailed description of the measurement technique [33].

## 4. Conclusions

A basic catalyst was created and utilized for catalyzing the aquathermolysis of heavy oil in this study. The viscosity of heavy oil was reduced by 88.3% using 0.2% B@Zn(II)L and 30% ethanol at 180 °C for 4 h. The heavy oil’s group composition was examined using GC, TGA, and DSC. The findings show that the reaction decreases the viscosity of the heavy oil and eliminates some heteroatoms from its molecules, enhancing flow characteristics and increasing quality. The GC-MS study shows that B@Zn(II)L can diminish the polar compounds in heavy oil and lower the aromaticity of these chemicals when dissolved in water. The GC-MS study of the thiophene and quinoline model compounds shows that the C-C, C-N, and C-S bonds are cleaved by the catalyst, resulting in the decreased viscosity of heavy oil.

## Figures and Tables

**Table 1:** The group composition of the oil sample before and after reaction.

Sample	Saturated HC %	Aromatic HC, %	Resin %	Asphaltene %
Blank	24.76	31.28	25.39	18.57
Oil + water	32.01	33.27	19.13	15.59
Oil + water + B@Zn(II)L	38.76	39.24	12.31	9.69
Oil + water + B@Zn(II)L + ethanol	39.97	40.06	11.03	8.94

**Table 2:** Chemical composition of heavy oil pre- and post-reaction.

Group	Composition, %					
	C	H	N	S	O	H/C
Blank	75.93	9.05	1.54	1.48	12.00	0.12
Oil + water	77.45	9.12	1.37	0.89	11.17	0.12
Oil + water + B@Zn(II)L	79.11	10.05	1.04	0.53	9.27	0.13
Oil + water + B@Zn(II)L + ethanol	81.18	11.04	0.99	0.46	6.19	0.14

**Table 3:** Characteristics of pores in B and B@Zn(II)L.

Name	Pore Size (nm)	Pore Volume (cm^3^/g)	Surface Area (cm^2^/g)
B	4.60	0.08	68.24
B@Zn(II)L	6.53	0.12	68.45

## Data Availability

The data presented in this study are available wholly within the manuscript.
